# From annotation to adaptation: extracting temporal relations in French clinical narratives

**DOI:** 10.1186/s12874-026-02867-4

**Published:** 2026-05-14

**Authors:** Judith Jeyafreeda Andrew, Juliette Potier, Nicolas Garcelon, Marc Vincent, Anita Burgun

**Affiliations:** 1 Clinical Bioinformatics Laboratory, INSERM UMR1163, Imagine Institute, Université Paris Cité, Paris, 75006 France; 2 PRAIRIE, PaRis Artificial Intelligence Research Institute, Paris, 75012 France; 3 Necker Enfants Malades Hospital, AP-HP, Paris, 75015 France

**Keywords:** Annotation guide, French clinical notes, Large language models, Temporal entity and Relation extraction

## Abstract

Extracting temporal information from unstructured clinical narratives is a foundational step toward automated patient timeline generation, a capability that has been proposed as having potential for rare disease diagnosis and care coordination, though prospective clinical validation remains future work. We present a comprehensive framework for temporal relation extraction from French clinical text, addressing a critical gap in non-English clinical NLP resources. We developed specialized annotation guidelines tailored to French medical language and created an annotated corpus of 490 clinical reports from Necker Hospital with 12,464 entity-relation pairs, achieving strong inter-annotator agreement (F1 $\ge$ 0.94 for core entities). Our comparative evaluation of modern AI approaches—including transformer-based models, large language models, and parameter-efficient fine-tuning (PEFT)-demonstrates that PEFT with CamemBERT-bio-base achieves the strongest temporal relation extraction performance (F1=0.82–0.87 for major relation types), significantly outperforming traditional approaches and matching few-shot large language models with greater computational efficiency. Entity consolidation substantially improves named entity recognition across all methods (DATE F1=0.96). This work provides validated temporal relation extraction methods as a technical foundation for future patient timeline generation systems. We discuss the pathway toward clinical integration, including deployment requirements, governance considerations, and the prospective validation studies needed to confirm clinical utility—particularly for rare genetic disease populations where automated temporal pattern recognition could support earlier diagnosis.

## Introduction

Clinical narratives in electronic medical records (EMRs) contain rich temporal information essential for reconstructing patient timelines and understanding disease progression, treatment responses, and clinical outcomes. However, extracting temporal information from unstructured clinical text and organizing it into coherent timelines remains challenging, requiring both robust annotation methodologies and effective computational approaches. Clinical documentation creates complex temporal networks spanning multiple time scales—from acute events documented in hours to chronic progressions tracked over years. These narratives involve diverse temporal expressions (precise timestamps like “le 15 mars 2023 à 14h30” (the 15th March 2023 at 14h30) versus vague references like *“quelques mois après l’apparition des premiers symptômes”(a few months after the onset of the first symptoms)*), complex relationships between medical entities (symptoms, procedures, interventions, outcomes), and causal dependencies where temporal precedence may indicate causation. Capturing these temporal complexities requires sophisticated natural language processing (NLP) approaches.

### Challenges and applications

Temporal relation extraction from clinical narratives faces several key challenges: (1) diverse temporal expressions requiring detection and normalization, (2) contextual ambiguity where temporal markers depend on surrounding context, (3) distinguishing temporal precedence from causal relationships, (4) handling progressive disease patterns and treatment responses, and (5) managing temporal uncertainty in retrospective documentation. Understanding temporal relationships enables critical clinical applications including automated patient timeline reconstruction for care coordination, disease progression analysis, diagnostic decision support through temporal pattern identification, treatment optimization, and clinical research facilitation.

Future Application to Rare Genetic Diseases: While this study uses general clinical narratives from Necker Hospital, the framework has particular relevance for rare genetic diseases. These conditions (affecting 3.5–5.9% of the global population [1] across over 7,000 distinct conditions [2]) present unique challenges including diagnostic delays averaging 5–7 years and sparse longitudinal data. Automated timeline generation could significantly reduce time to diagnosis by revealing characteristic temporal patterns and support monitoring of disease evolution over decades.

Clinical Impact: Automated temporal extraction has been proposed to support critical clinical applications, contingent on future pipeline development and prospective validation: (1) reducing diagnostic delays in rare diseases by revealing characteristic temporal patterns across fragmented records, (2) supporting clinical decision-making through automated timeline visualization that highlights disease progression and treatment responses, and (3) enabling retrospective cohort studies by structuring longitudinal data currently locked in narrative text. For rare genetic diseases specifically, automated timeline generation could reduce time to diagnosis by making temporal patterns immediately visible to clinicians [3].

### Study contributions and scope

This study addresses temporal relation extraction for patient timeline generation in French clinical narratives by developing and evaluating novel NLP approaches. Using clinical narratives from Necker Hospital, our contributions include:

- Annotation Guideline for Timeline Generation: We develop a comprehensive annotation guideline specifically designed for temporal entities and relations relevant to patient timeline construction, addressing the specific requirements of chronological event ordering and causal relationship identification in clinical texts.
- Annotated Dataset: We create an annotated dataset of French clinical narratives with temporal entities and relations for timeline generation, and report key insights from its analysis.
- Comparative Methodology Evaluation: We compare modern approaches including large language models (LLMs) and parameter-efficient fine-tuning (PEFT) methods against established toolkits (OpenNRE, FLAIR) for temporal entity recognition and temporal relation extraction, providing empirical evidence for model selection in clinical timeline generation tasks.
- Performance Analysis of LLMs vs.PEFT vs. Traditional Frameworks: We provide comprehensive evaluation demonstrating how prompting LLMs and PEFT strategies with transformers compare to specialized NLP toolkits, offering insights into trade-offs between generalist and specialist approaches for clinical temporal relation extraction.
- Automated Timeline Generation Framework: We present the foundational relation extraction components required for patient timeline generation and discuss the pathway toward a complete automated system, which remains future work.

This work provides a comprehensive investigation of temporal relation extraction in French clinical documentation for timeline generation, contributing both methodological innovations through comparative evaluation of modern approaches and practical clinical applications. While developed on general clinical narratives, the framework establishes foundational capabilities for future application to specialized contexts such as rare genetic diseases, where automated temporal information extraction could address pressing clinical needs in diagnosis and disease management.

While our methodology using clinical narratives from Necker Hospital, the framework is designed for generalizability. The annotation guidelines are language-specific (French) rather than institution-specific, the entity taxonomy captures universal temporal concepts in clinical documentation, and the comparative evaluation framework (FLAIR, OpenNRE, LLMs, PEFT) is reproducible across institutions. Institution-specific elements are limited to vocabulary and documentation style, which standard domain adaptation techniques can address.

## Related Work

### Temporal relation extraction in clinical texts

Temporal relation extraction (TRE) has emerged as critical for reconstructing patient timelines from unstructured medical texts. Recent systematic reviews underscore the dominance of transformer architectures in TRE tasks, particularly for English-language corpora [4]. Han et al. [5] introduces OpenNRE, providing a unified framework for neural relation extraction with pre-trained models and benchmarks. HECTA-UoM, MedTem [6] provide a toolkit for medication and treatment temporal modelling. However, significant gaps remain in multilingual resources and domain-specific datasets [7]. Kougia et al. [8] present the first comprehensive zero-shot TRE study for biomedical text, demonstrating generalization potential without extensive domain training while Yuan et al. [9] show a complementary study on zero-shot temporal relation extraction using LLMs in biomedical text. Cui et al. [10] have presented a prompt-based temporal classification of treatment events showing promising results.

### Multimodal and knowledge-enhanced approaches

Recent work explores multimodal and retrieval-augmented methods to address traditional approach limitations. Knez and Žitnik [11] propose a bimodal transformer integrating textual features with knowledge graph embeddings, improving capture of sparse temporal cues. Zhang et al. [12] introduce retrieval-augmented prompting for LLMs, enhancing temporal relation classification through external knowledge. These innovations demonstrate that combining structured knowledge with neural representations significantly enhances TRE accuracy in complex clinical narratives.

### French clinical NLP and language-specific models

French clinical NLP has advanced through specialized models. Bannour et al. [13] reframe TRE as sequence labeling relative to document creation time, achieving strong performance on oncology reports. DrBERT [14] represents the first robust French biomedical pre-trained model, while Bazoge et al. [15] and AliBERT [16] addresses French long documents. Le et al. [17] tackle computational challenges through Mixture of Experts approaches. However, biomedical models require fine-tuning for downstream tasks, raising privacy concerns for cross-institutional sharing. Andrew et al. [18] and Andrew et al. [19] demonstrated competitive few-shot prompting with LLMs for French pediatric clinical texts.

### Transformer models and PEFT in biomedical applications

Biomedical transformers (BioBERT [20], MedBERT [21]) have shown substantial improvements over general-purpose models. Akbik et al. [22] introduces Flair, offering contextual string embeddings and transformer integration for sequence labeling. Parameter-Efficient Fine-Tuning (PEFT) has emerged as promising for domain adaptation. Pu et al. [23] and Zhou et al [24] demonstrate PEFT methods (LoRA, Prefix-Tuning, Adapters) maintain performance while reducing trainable parameters, particularly effective for classification tasks. Li and Liang [25] show prefix-tuning matches full fine-tuning with fewer parameters, while [26] demonstrate prompt tuning viability at scale. These establish foundations for efficient, domain-adaptable temporal relation extraction.

### Methodological gaps

Despite progress, challenges persist: data scarcity for non-English languages, limited generalization across clinical domains, difficulty with long-range temporal dependencies, and lack of standardized evaluation frameworks. This work addresses these gaps by developing comprehensive annotation guidelines for French clinical texts, comparing modern PEFT approaches against traditional frameworks and LLMs, and establishing methodologies for patient timeline generation in low-resource clinical contexts.

## Temporal annotation guidelines for clinical reports

### Overview and objectives

The temporal annotation framework was developed to systematically capture temporal relationships between clinical phenotypes and time expressions in French clinical reports. This builds on existing works like TimeML [27] and MERLOT [28]. The primary goal is to create a comprehensive temporal representation that links each phenotypic mention with relevant temporal entities, whether precise or approximate, to establish a complete timeline of medical events. The guidelines address the inherent complexity of temporal expressions in clinical discourse, which often involve implicit references, relative timing, and varying levels of precision that require careful linguistic and contextual analysis.

The annotation process produces three types of annotations: entities (text spans identifying temporal or phenotypic expressions with specified types), attributes (additional information associated with entities beyond their basic type), and relations (directed or undirected links between annotated entities). This multi-layered approach enables the capture of both explicit temporal information and the complex inferential relationships that characterize clinical temporal reasoning.

Our framework builds on, but deliberately extends, two key prior resources. TimeML [27] and its ISO standardisation (ISO-TimeML) define TIMEX3 expressions (DATE, TIME, DURATION, SET) and TLINK relations (BEFORE, AFTER, INCLUDES, IS_INCLUDED, SIMULTANEOUS, BEGINS, BEGUN_BY, ENDS, ENDED_BY) for general event-temporal annotation. Our entity taxonomy inherits this broad structure but introduces clinically motivated subtypes absent from TimeML: the DATE class is decomposed into six subtypes (DOB, DOR, DOV, DOPV, DOFV, DOTHER) with corresponding _incomplete variants, reflecting the distinct temporal anchoring roles these categories play in patient-timeline construction.

MERLOT [28] provides a French clinical corpus with semantic annotations including temporal entities, and was an important reference for French clinical temporal expression patterns. However, MERLOT does not provide a relation schema oriented toward patient timeline generation; our work therefore extends the MERLOT entity layer with a richer relation taxonomy suited to chronological patient-event ordering. Additionally, unlike MERLOT’s general hospital text, our corpus is drawn from a specialized pediatric rare-disease setting, introducing unique temporal expression patterns (gestational ages in SA units, disease-onset relative expressions such as “depuis l’âge de 12 ans”) not covered by prior French clinical annotation frameworks.

### Temporal entity classification framework

#### Definitional categories

The annotation framework establishes a comprehensive taxonomy that distinguishes between different types of temporal entities based on their precision and reference frame. Time expressions are classified along three primary dimensions that guide annotation decisions. First, the distinction between absolute and relative temporal entities helps determine entity subclasses, where absolute expressions refer directly to specific dates or times (such as *“24 mai 2004”(24 May 2004)* or *“14H”*), while relative expressions depend on other temporal references for interpretation (such as *“deux jours après l’opération”(two days after the operation)* or *“J2”(Day 2)*. These relative expressions include, among others, all time entities whose time reference is determined according to when the report was written, such as *“ce jour”(this day)*. Second, the precision distinction between precise and approximate expressions influences relation type selection, with precise expressions providing quantifiable temporal distances and approximate expressions indicating imprecise temporal spans like *“récent”(recent)*, *“il y a une dizaine de jour”(about ten days ago)*, or *“4 à 5 jours”(4 to 5 days)*. Third, the framework distinguishes between time points (single moments in time) and durations (spans between two time points), which affects both entity classification and relationship annotation.

#### Entity type specifications

##### DATE Entities

DATE entities encompass various clinical timepoints that serve as temporal anchors within medical reports. The framework defines six distinct subtypes based on their clinical significance and temporal reference. DOB (Date of Birth) entities capture birth dates for any person mentioned in the text, including patients, relatives, or donors, such as references like *“née le 15/03/1985”(born on the 15/03/1985)*. DOR (Date of Report) entities identify when clinical reports were created, dictated, edited, printed, or validated, including dates of multidisciplinary team meetings (RCP dates). DOV (Date of Visit) entities mark current consultation, examination, or hospitalization dates, including *“date d’entrée”(date of entry)*, *“date de sortie”(date of exit)*, or specific examination dates. DOPV (Date of Past Visits) entities capture historical medical encounters referenced in sections like *“ANTECEDENTS”(Past Medical History)*, while DOFV (Date of Future Visits) entities identify scheduled future appointments or procedures typically found in Conclusion sections. DOTHER entities encompass any other dates present in clinical text, including historical diagnoses, treatment dates, or significant medical events.

The framework distinguishes between complete and incomplete date entities based on year specification. Complete dates include year information (optionally with month and/or day), while incomplete dates lack year specification and are marked with “_incomplete” suffixes (DOB_incomplete, DOR_incomplete, etc.). For example, *“Craniopharyngiome type découvert sur des signes d’HTIC en Août”(Craniopharyngioma type discovered based on signs of intracranial hypertension in August)* would be annotated as DOTHER_incomplete due to the missing year, while *“Début de Diabète: 03.2015”(early signs of diabetes and Diabetes onset: 03.2015)* represents a complete DOTHER entity despite lacking day specification.

##### TIME Entities

TIME entities capture relative temporal expressions that reference specific time points without providing absolute dates. These range from precise quantified expressions like *“4 semaines”(4 weeks)* and *“T-30min”* to approximate temporal references such as *“24-48 heures”* and *“mois suivant”(the following month)*. The annotation includes contextual phrases that embed time points, such as *“jour de l’examen”(date of the exam)* or *“veille de l’examen”(the day before the exam)* but excludes isolated event terms like *“examen”(exam)* or *“traumatisme”(trauma)* which are classified separately as events.

Specific annotation rules govern TIME entity boundaries. Quantified time expressions exclude prepositions and postpositions, like *“à bientôt 3 ans de”(almost 3 years after)*, or *“il y a deux mois”(two months ago)* are annotated as *“bientôt 3 ans”(almost 3 years)* and *“deux mois”(2 months)* respectively. However, prepositional phrases that embed time points with events are annotated inclusively, such as *“après un AVC”(after a stroke)* or *“à la naissance”(at birth)*. Temporal adjectives like *“anténatal”(prenatal)* and *“néonatal”(neonatal)* are included as they refer to approximate time points with clinical significance.

##### AGE Entities

AGE entities mark any age references within clinical text, encompassing patient ages, family member ages, donor ages, fetal ages, or even bone ages, like *“quinze ans”(Fifteen years old)*. Fetal ages are typically expressed in gestational terms such as *“SA”* (semaines d’aménorrhée (weeks of amenorrhea)) or *“Age Gestationnel”(Gestational Age)*. When ages are expressed in days or months, fragmented annotations capture both numerical and unit components to maintain precision.

##### DURATION Entities

DURATION entities identify continuous time spans that either explicitly mention temporal length or define periods through start and end points. Examples include explicit durations like *“pendant 2 jours”(for 2 days)* or *“sur 3 heures”(over 3 hours)*, as well as implicit durations defined by temporal boundaries such as *“depuis le 20/01/2001”(since the 20/01/2001)* or *“d’ici là”(until then)*. Duration annotations include qualifying terms like *“depuis”(since)*, *“pendant”(during)*, or *“à partir de mai 2010”(from May 2010)* to capture the complete temporal expression.

For durations defined by start and end points, the framework requires multiple annotations: separate entities for each time point, a DURATION entity for the complete expression, and a duration relation linking the time points. This approach enables both temporal ordering through relations and duration-specific temporal relationships with phenotypes.

##### FREQUENCY Entities

FREQUENCY entities capture recurring temporal patterns and regular intervals. These include medication dosing schedules like *“par jour”(per day)* or *“tous les matins”(every morning)* and clinical visit schedules like *“tous les 8 jours”(every 8 days)*. Frequency annotations also encompass abbreviated forms common in medical contexts, such as *“2x/j”(2x/day)* which are annotated as complete expressions.

##### EVENT Entities

EVENT entities serve as temporal anchors when explicit time expressions are unavailable in clinical reports. These are annotated only when necessary to establish temporal relations, capturing relevant clinical events like *“examen”* or *“bilan”(balance sheet)*. Event selection prioritizes clinical relevance and the nature of the report, with annotations excluding articles to maintain consistency.

### Temporal relationship framework

#### Phenotype-to-time relations

The framework establishes six primary relation types that capture different temporal associations between clinical phenotypes and temporal entities. The begins-at relation indicates when a phenotype starts at a specified time point or within a duration period, used when temporal onset is clearly established, as in linking symptom emergence to specific dates or connecting hypothetical phenotypes to the dates when hypotheses are proposed. The ends-at relation marks phenotype cessation at particular time points, particularly useful for linking conditions to resolution dates or surgical interventions that terminate phenotypes.

The before relation establishes temporal precedence when phenotypes end before referenced time points or duration beginnings, while the before-overlap relation captures conditions that predate but continue during specified time periods. This relation is particularly applicable to chronic conditions, examination findings that reveal pre-existing phenotypes, or situations where phenotype duration is uncertain but ongoing presence is confirmed.

The simultaneous relation indicates shared timespans between phenotypes and temporal entities, used when exact temporal correspondence is established, such as linking intermittent phenotypes with their frequency patterns or connecting phenotypes with their known durations. The overlap relation represents partial temporal correspondence when precise timing relationships are uncertain, particularly applicable when temporal entities refer to approximate times or when phenotype durations are unclear.

The six phenotype-to-time relation types constitute a clinically motivated coarsening of Allen’s interval algebra [29], which defines 13 mutually exclusive base relations between temporal intervals. The mapping is as follows: BEGINS-AT corresponds to Allen’s starts relation (phenotype onset coincides with the start of a time span); ENDS-AT to Allen’s finishes (phenotype termination coincides with a time point); SIMULTANEOUS to Allen’s equals (phenotype and time span share identical boundaries); OVERLAP to Allen’s overlaps and during combined (partial temporal correspondence, used when precise boundaries are unavailable); BEFORE to Allen’s before and meets (phenotype entirely precedes the time reference); and BEFORE-OVERLAP to a composite covering Allen’s overlaps and starts variants where the phenotype is established prior to but continues into the referenced period—a pattern highly characteristic of chronic conditions in clinical documentation. Two Allen relations, met-by and finished-by, are not represented as primary relations in our schema because their clinical expression is typically handled through the inverse-direction Time-to-Phenotype annotations. This coarsening was motivated by two factors: (1) the level of temporal precision available in retrospective clinical narratives rarely supports distinguishing all 13 Allen relations reliably, and (2) the six selected relations capture all patterns needed for forward chronological timeline assembly.

#### Time-to-time relations

Temporal relationships between time expressions are annotated selectively, only when they provide additional information for existing phenotype-time relationships or disambiguate temporal entities. For instance expressions such as *“ce jour”(this day)*, would be only linked to a DOV or a DOR only if there is several DOV or DOR in the report. This is to disambiguate while maintaining annotation simplicity. The duration relation links start and end time points to establish temporal spans, used in conjunction with DURATION entities to capture complete temporal ranges. The simultaneous relation between temporal entities disambiguates different expressions that refer to identical temporal references, such as linking *“J15’(Day 15)’* and *“J3”(Day 3)* or connecting *“20/01”* with *“J4”(Day 4)* when they represent the same timepoint.

The contains relation indicates when one temporal entity encompasses another, typically used when a duration contains a specific time point that has phenotypic relationships. The equivalent relation serves as an annotation shorthand when multiple time expressions share identical phenotypic relationships, improving annotation readability by avoiding crossing relation lines while maintaining complete temporal information.

#### Phenotype-to-phenotype relations

Phenotype-to-phenotype relations are annotated only when one phenotype already has temporal relationships established. The primary relation type is **equivalent**, which serves as an annotation shorthand for phenotype sets sharing identical temporal information. This notation improves annotation efficiency and readability by using a phenotype-time relationship as reference (involving the phenotype closest to the time entity) and chaining consecutive phenotypes sharing the same relation to the time entity with the aforementioned equivalent relation type. This avoids redundant crossing annotations while preserving complete temporal relationship information.

### Annotation rules and complex constructions

#### Embedded entities and ranges

The framework addresses complex temporal constructions through specific annotation protocols. Embedded entities, such as *“depuis août 2010”(since August 2010)*, *“depuis l’âge de 12 ans”(since the age of 12 years)*, or *“du 15 au 25 avril 2016”(from 15 to 25 April)*, require annotation of both embedding and embedded temporal expressions. Temporal ranges necessitate separate annotation of constituent elements with subsequent duration relations linking them. For example, *“hospitalisée dans le service du 22 au 29 avril 1997”(hospitalized in the ward from April 22nd to 29th, 1997)* requires fragmented annotation of *“22 avril 1997”(22 april 1997)* and *“29 avril 1997”(29 april 1997)* as separate DOV entities, connected by a duration relation.

#### Incomplete dates and contextual resolution

Incomplete date handling follows systematic rules based on temporal precision and contextual information. Dates lacking year specification receive “_incomplete” suffixes, while contextual completion may be performed when temporal information allows unambiguous resolution. For instance, *“Je propose un rendez-vous de consultation le 20 décembre”(I propose a consultation appointment on December 20th.)* would be annotated as DOFV_incomplete despite including day and month information due to missing year specification.

#### Attribute integration and negation handling

The framework incorporates phenotypic attributes that affect temporal relationship annotation. Negated phenotypes (marked with ‘is_negated’ -as a result of the automated phenotypic pre-annotation- or ‘annotator_negated’, when a correction by the annotator was needed) maintain temporal relations that describe when phenotype absence was established or confirmed. Hypothetical phenotypes (marked with ‘hypothesis’) are linked to times when hypotheses were proposed. Additional attributes like ‘increasing’, ‘decreasing’, ‘potential’, and ‘SpanNotSure’ provide contextual information that may influence temporal relationship selection and interpretation.

#### Annotation priority and entity selection

When multiple overlapping phenotypic annotations exist from different recognition methods (UMLS-based and deep learning-based), selection follows temporal information preservation principles. The annotation prioritizes spans that maintain the most temporal information, preferring longer spans when temporal content is equivalent, and defaulting to deep learning annotations when spans are identical in length and information content. This approach ensures optimal temporal relationship annotation while maintaining compatibility with automated recognition systems.

## Dataset Statistics and Analysis

### Corpus overview

The annotated corpus comprises 490 clinical reports with a total of 12,464 entity-relation pairs, yielding an average of 25.44 pairs per document.

### Entity type distribution

**Fig. 1:**
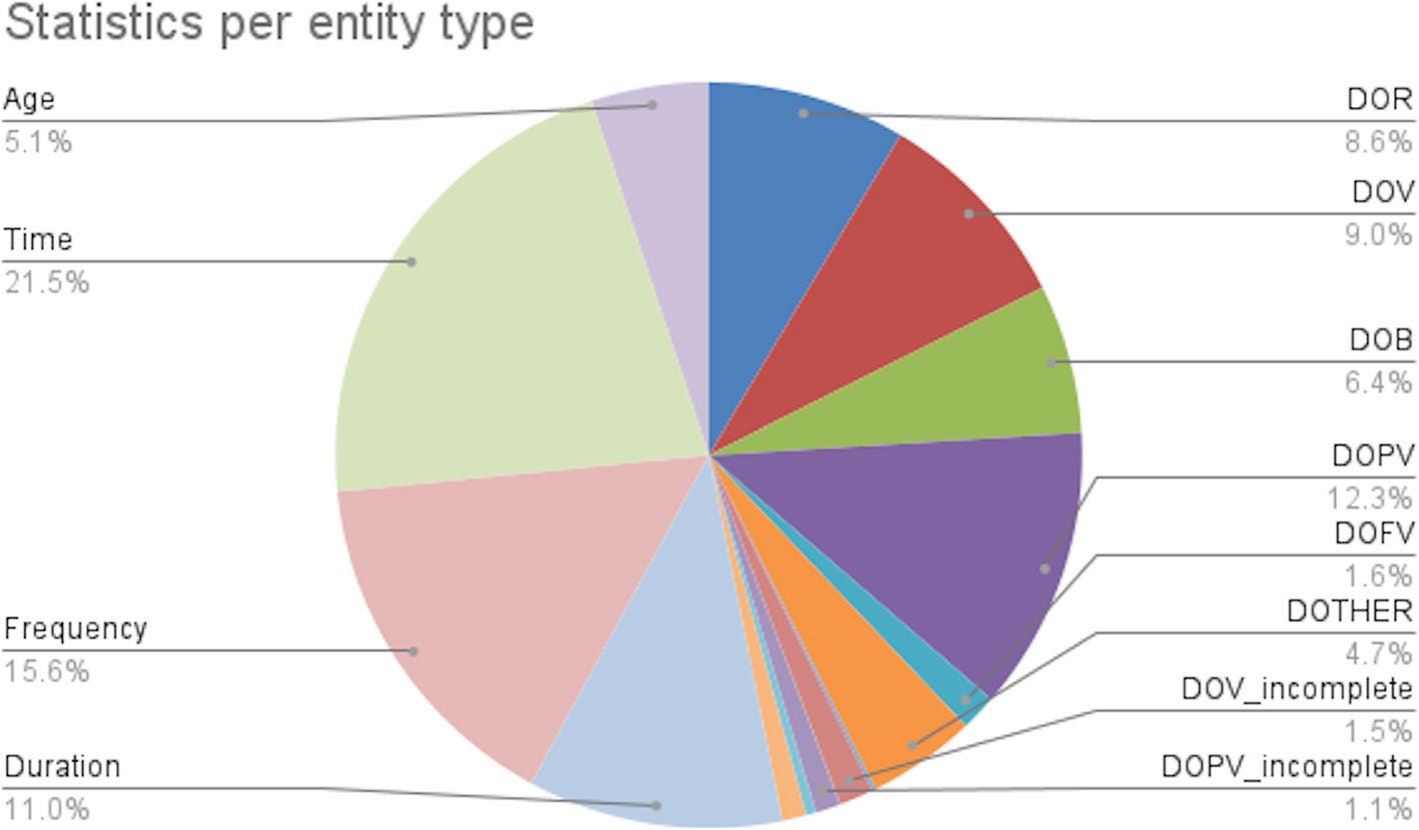
Distribution of temporal entity types across the 490-document corpus. DATE subtypes (Date of Birth (DOB), Date of Report (DOR), Date of Visit (DOV), Date of Past Visit(DOPV), Date of Future Visit(DOFV), Other Dates (DOTHER) and their _incomplete variants) collectively account for approximately 40% of all annotated temporal entities, making them the dominant category; TIME expressions (21.5%) are the most frequent single non-DATE entity type

Figure 1 and Table 1 show the distribution of the temporal entities within the corpus.

**Table 1:** Distribution of temporal entity types in the annotated corpus

Entity type	Count
TIME	1162
FREQUENCY	842
DURATION	598
AGE	276
DATE (all subtypes)	2536
DOB	348
DOV	488
DOPV	667
DOTHER	255
DOR	465
DOFV	85
DOB_incomplete	1
DOV_incomplete	80
DOPV_incomplete	58
DOTHER_incomplete	56
DOR_incomplete	9
DOFV_incomplete	24

Frequent entities such as *“ce jour”* and *“actuellement”* reflect the temporal entities commonly used in clinical documentation. The prominence of *“post-operatoire”* and age markers like *“3 ans”* underscores the dataset’s relevance to pain tracking and pediatric care.

### Temporal relationship patterns

**Fig. 2:**
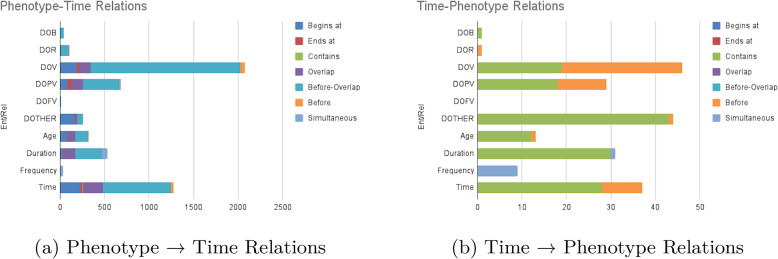
Distribution of temporal relation types between Phenotype and Time entities across the annotated corpus. **a** Phenotype → Time relations: counts of each relation type (BEGINS-AT, ENDS-AT, CONTAINS, OVERLAP, BEFORE-OVERLAP,BEFORE, SIMULTANEOUS) broken down by target temporal entity type (DOB, DOR, DOV, DOPV, DOFV, DOTHER, AGE, Duration, Frequency, Time). DOPV is the dominant temporal anchor, reflecting the retrospective nature of the clinical narratives, where most phenotypes are documented as historical findings relative to a past medical encounter. BEFORE-OVERLAP is the most frequent relation type across virtually all entity types. **b** Time → Phenotype relations: the mirror perspective, showing counts of each relation type broken down by source temporal entity type. BEFORE-OVERLAP again dominates across all temporal entity types in both directions, confirming that chronic and ongoing conditions — which predate but persist through the documented time reference — constitute the canonical temporal pattern in this pediatric rare-disease corpus

Table 2 shows the distribution of the relation types across the corpus. The presence of nuanced relations like BEFORE-OVERLAP and BEGINS-AT highlights the corpus’s capacity to capture chronicity and the onset of symptoms - crucial for longitudinal patient modeling. The Figs. 2a and b show fine-grained statistics regarding the number of relation types per entity type with different source and target entities.

**Table 2:** Relation type distribution

Relation	Count	Percentage
BEFORE-OVERLAP	6,465	51.87%
BEGINS-AT	2,317	18.59%
OVERLAP	1,555	12.48%
CONTAINS	801	6.43%
BEFORE	438	3.51%
SIMULTANEOUS	352	2.82%
DURATION	320	2.57%
ENDS-AT	216	1.73%

### Inter-annotator agreement

Annotation setup: Annotations were performed using the brat rapid annotation tool [30], configured with our custom entity and relation schema. Two annotators performed the annotation of the full 490-document corpus: one annotator has a background in computational linguistics with text annotation experience; the second has a background in clinical informatics with experience with experts to create evaluation metrics. Disagreements during the main annotation phase were resolved by discussion between the two annotators, with a third senior author as arbiter for unresolved cases.

Pre-annotation: Phenotypic entity spans were automatically pre-annotated using the two complementary methods described in Phenotypes pre-annotation section. This approach automatically detected phenotype spans served as fixed anchors for temporal annotation; annotators were asked to identify temporal entities and temporal relations but were not asked to modify or validate phenotype spans.

Inter-annotator agreement: Inter-annotator agreement was computed on a held-out subset of 50 documents (approximately 10% of the corpus), independently annotated by both annotators from scratch without cross-revision. Entity agreement was computed using strict span matching: a predicted entity span is counted as correct only if both the character-level boundaries and the entity type match exactly between the two annotators; no partial-overlap credit was given. Relation agreement was computed as follows: a relation instance counts as matching if both annotators identified identical source entity spans, identical target entity spans (strict matching on both), and the same relation type. No temporal closure was applied. Precision, Recall and F1 were computed per entity/relation type using micro-averaging across all annotated instances in the IAA documents.

The inter-annotator agreement evaluation was conducted from scratch, with annotators provided only with pre-identified phenotypes. This approach captures the cumulative challenge of the complete annotation pipeline, including: (1) identifying temporal entity spans and types, (2) determining temporal relation existence between entities, and (3) classifying the specific relation type. The reported agreement scores therefore reflect real-world annotation difficulty across all these decision points.

Temporal Entity Agreement: Table 3 shows strong inter-annotator agreement across temporal entities. AGE and DATE entities achieved perfect agreement (F1=1.00), reflecting their unambiguous nature. Duration (F1=0.96), Frequency (F1=0.95), and Time entities (F1=0.94) also demonstrated excellent consistency, with minor disagreements primarily in boundary detection for approximate temporal expressions.

**Table 3:** Inter annotator agreement (IAA) for temporal entities

Entity	Precision	Recall	F1 Score
AGE	1.00	1.00	1.0000
DATE	1.00	1.00	1.0000
Duration	0.98	0.94	0.9596
Frequency	0.93	0.97	0.9496
Time	0.96	0.93	0.9448

Temporal Relation Agreement: Table 4 presents agreement scores for temporal relations. BEFORE-OVERLAP achieved the highest agreement (F1=0.94; precision=1.00, recall=0.88), followed by BEGINS-AT (F1=0.92) and ENDS-AT (F1=0.90). BEFORE (F1=0.85) and CONTAINS (F1=0.81) relations showed good agreement. SIMULTANEOUS (F1=0.75) and OVERLAP (F1=0.71) exhibited lower agreement, reflecting the subtle semantic distinctions between these relation types and the challenges of interpreting approximate temporal correspondences in clinical narratives. Overall, the IAA results demonstrate that our annotation guidelines produce reliable and consistent annotations, with particularly strong agreement on core temporal entities and high-frequency relation types essential for timeline generation.

**Table 4:** Inter annotator agreement (IAA) for temporal relations

Relation	Precision	Recall	F1 Score
BEGINS-AT	0.95	0.90	0.9243
ENDS-AT	0.90	0.90	0.9000
CONTAINS	0.87	0.75	0.8056
BEFORE	0.92	0.79	0.8501
OVERLAP	0.88	0.60	0.7135
BEFORE-OVERLAP	1.00	0.88	0.9362
SIMULTANEOUS	0.70	0.80	0.7467

## Experiments

For our experiments, we use a split of our dataset with 273 files for training, 117 files for validation and 100 files for testing. Our experiments reflect a pipeline architecture where we use the raw text marked with phenotypes to identify temporal entities. The phenotypes and temporal entities are then used as inputs along with the text to identify the relation between them.

For the purpose of temporal entity detection, we use FLAIR [22] and compared its performance with zero shot and few shot prompting on two LLMs. The entity recognition is evaluated in two ways, firstly using all the fine grained entity types mention in Temporal entity classification framework section and secondly by consolidating all the DATE entities into one single class.

Following this, for the purpose of temporal relation extraction we present a two step approach where the first step is to identify a presence or absence of relation given all possible entity pairs. This is followed by a multi-class classification step where the models are provided with the entity pairs that are sure to have a relation and are asked to identify the relation based on the annotation guidelines discussed in Temporal annotation guidelines for clinical reports section. Figure 4 (in Appendix section) shows the overview of the pipeline used for this experimentation.

All experiments were performed in a secure, privacy-preserving environment to ensure the confidentiality of clinical documents. No external API calls or cloud-based inference were used; all processing was conducted locally within a controlled infrastructure compliant with data protection standards. This setup was essential given the sensitive nature of the French clinical narratives used in this study.

Before any experiment clinical notes are automatically anonymized and preprocessed with pre-existing methods to remove protected health information and formatting artifacts.

### Named entity recognition

We conducted experiments comparing different frameworks and approaches for the purpose of temporal Named Entity Recognition: deep learning on one hand - using the FLAIR library and LLMs with zero and few-shot prompting on the other.

#### Phenotypes pre-annotation

Phenotypes were automatically pre-annotated using pre-existing methods. Two complementary methods were used. The first one is based on a dictionary approach to phenotype mentions detection followed by a deep learning approach to context detection (including filtering non relevant mentions and determining negated mentions) [31]. The second one is an extension of this work with mention detection directly performed by deep learning. The latter completes the former with out-of-vocabulary or longer mentions. The detected phenotypes were used as anchors for the temporal relations during the annotation process.

#### Temporal entities

##### FLAIR

To support the extraction of temporal entities from French clinical narratives, we use the FLAIR NLP framework.

Input texts were tokenized using Flair’s default tokenizer for CamemBERT. Labels were mapped to Flair’s Label objects for multi-class classification. We have performed NER experiments in two iterations. In the first iteration, all categories mentioned in Entity type specifications section are used, while in the second iteration the DATE categories (DOB, DOR, DOV, DOPV, DOFV, DOTHER and their “_incomplete” couterparts) are collapsed into one label as “DATE”. The results of both evaluations are shown in Table 5, where the fine-grained entities are listed below the consolidated entity DATE.

**Table 5:** Precision (P), Recall (R), and F1 scores for temporal named entity recognition across all models and entity types

	FLAIR			LLaMA Zero-shot			Mistral Zero-shot			LLaMA Few-shot			Mistral Few-shot		
**Entity**	P	R	F1	P	R	F1	P	R	F1	P	R	F1	P	R	F1
Age	0.847	0.808	0.827	0.420	0.360	0.388	0.460	0.400	0.428	0.530	0.500	0.474	**0.600**	**0.550**	**0.574**
DATE (consolidated)	**0.958**	**0.967**	**0.963**	0.700	0.670	0.685	0.780	0.810	0.795	0.830	0.850	0.840	0.910	0.900	0.905
*Fine-grained DATE subtypes*															
DOB	**0.975**	**0.989**	**0.982**	0.890	0.900	0.895	0.880	0.840	0.860	0.910	0.900	0.884	0.930	0.900	0.915
DOFV	**0.704**	**0.746**	**0.725**	0.620	0.600	0.610	0.680	0.600	0.638	0.720	0.630	0.676	0.740	0.700	0.719
DOFV_incomplete	0.206	0.200	0.203	0.050	0.100	0.067	0.120	0.080	0.096	0.170	0.200	0.123	**0.200**	**0.220**	**0.210**
DOPV	0.606	0.798	**0.689**	0.380	0.400	0.390	0.420	0.440	0.430	0.580	0.420	0.494	0.620	0.540	0.577
DOPV_incomplete	0.483	0.127	0.202	0.040	0.070	0.051	0.150	0.090	0.113	0.170	0.200	0.135	**0.230**	**0.200**	**0.214**
DOR	**0.936**	**0.970**	**0.952**	0.550	0.570	0.560	0.470	0.500	0.485	0.620	0.660	0.544	0.710	0.650	0.679
DOR_incomplete	N/A			N/A			N/A			N/A			N/A		
DOTHER	0.795	0.299	0.434	0.640	0.580	0.609	0.680	0.620	0.649	0.720	0.680	0.682	**0.820**	**0.780**	**0.800**
DOTHER_incomplete	N/A			N/A			N/A			N/A			N/A		
DOV	**0.832**	**0.739**	**0.783**	0.440	0.390	0.414	0.530	0.500	0.515	0.540	0.430	0.527	0.650	0.580	0.613
DOV_incomplete	0.381	0.703	**0.494**	0.030	0.070	0.042	0.050	0.080	0.062	0.130	0.110	0.084	0.180	0.200	0.190
Duration	**0.835**	**0.733**	**0.780**	0.580	0.530	0.554	0.590	0.550	0.569	0.700	0.670	0.628	0.720	0.690	0.705
Frequency	**0.870**	**0.791**	**0.829**	0.540	0.520	0.530	0.580	0.540	0.559	0.570	0.610	0.565	0.620	0.570	0.594
Time	**0.796**	**0.685**	**0.736**	0.590	0.520	0.553	0.530	0.500	0.515	0.690	0.640	0.590	0.680	0.590	0.632

Best F1 per row in **bold**. A horizontal rule separates the consolidated DATE evaluation (top) from fine-grained DATE subtype evaluation (below)

***Model Architecture:*** We fine-tuned the camembert-base model for token-level sequence labeling using Flair’s SequenceTagger module with TransformerWordEmbeddings. Input tokens are embedded using contextual representations from CamemBERT, and the sequence tagger applies a linear CRF layer over the token-level embeddings to predict BIO-tagged entity spans. This is the standard Flair formulation for named entity recognition and produces per-token label predictions that are decoded into entity spans. Training Configuration followed Flair’s default setup: Loss Function: CrossEntropyLoss, Optimizer: AdamW, Learning Rate: 5e-5, Batch Size: 32, Max Epochs: 10.

##### Large Language Models

To evaluate the performance of LLM in Named Entity Recognition task, we chose two high-performing open-source transformer models: LLaMA 8B and Mistral-small 22B.

*Zero shot prompting:* For the zero shot approach, the model given the task and simply asked to perform the task relying on its pre-training. The evaluation is two folds, one fine-grained entities and the other for consolidated DATE entities. The prompt for the fine-grained entity recognition task is shown below, titled ’zero-shot Temporal Entity Recognition Prompt’. For the evaluation with consolidated DATE entities, we collapse all DATE entities such as DOB, DOR, DOV, DOPV, DOFV, DOTHER and their *_incomplete counterparts into one entity - DATE. The results both the evaluation is shown in Table 5, where the fine-grained entities are listed below the consolidated entity DATE. 
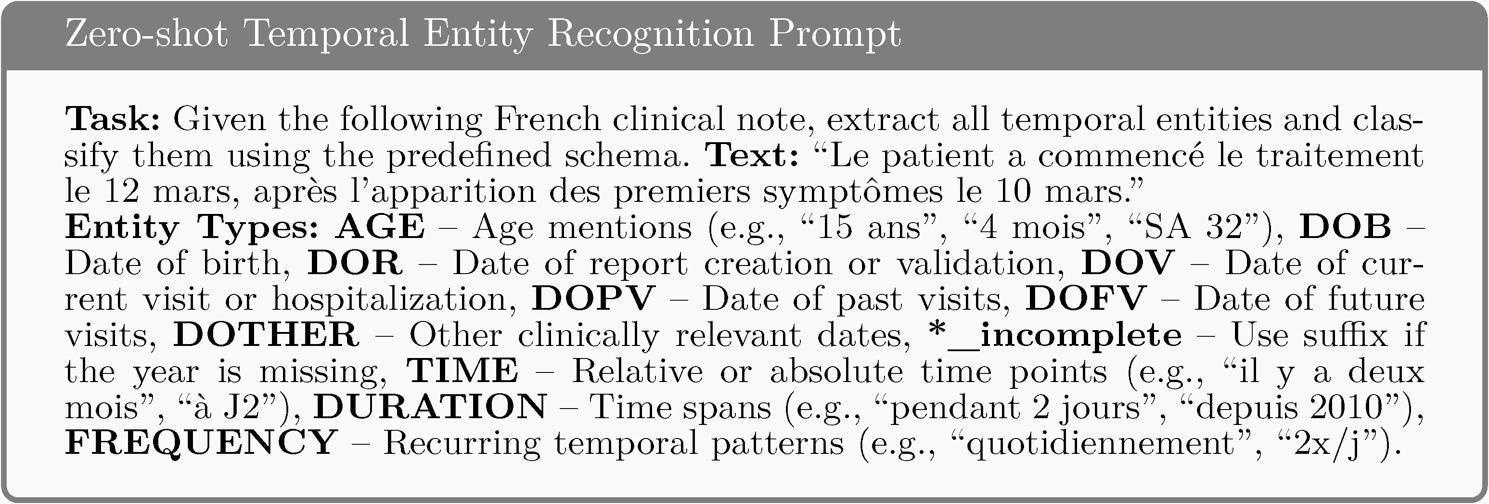


*Few shot prompting:* The few shot prompts allows the LLM to understand the task with the help of a few examples. The evaluation for this approach is also performed in two folds, one fine-grained entities and the other for consolidated DATE entities. The prompt for the fine-grained entity recognition task is shown below, titled ’few-shot Temporal Entity Recognition Prompt’. For the evaluation with consolidated DATE entities, we collapse all DATE entities such as DOB, DOR, DOV, DOPV, DOFV, DOTHER and their *_incomplete counterparts into one entity - DATE, just as the experiments with zero-shot prompting and FLAIR.

Table 5 summarises NER performance across all models and entity types; the best F1 per row is shown in bold. FLAIR achieves the strongest performance overall, leading on 12 of 16 entity types, while few-shot Mistral-small 22B performs best on Age and the rarer DATE subtypes. N/A rows (DOR_incomplete, DOTHER_incomplete) indicate near-zero test-set support where metric estimates are unreliable. Rows marked N/A (DOR_incomplete, DOTHER_incomplete) indicate entity types with near-zero test-set instances (fewer than 5 occurrences) where precision, recall, and F1 estimates are unreliable and are therefore suppressed rather than reported as zero. For all other rows, F1 values are computed as the harmonic mean of the displayed precision and recall; apparent minor rounding discrepancies (e.g., displayed P=0.483, R=0.127 yielding F1=0.202) reflect rounding of the displayed figures to three decimal places while F1 is computed from unrounded values. Confidence intervals were not computed for individual entity and relation types due to the single test-set evaluation design; future work should employ bootstrap resampling or cross-validation to provide robustness estimates, particularly for low-support categories. 
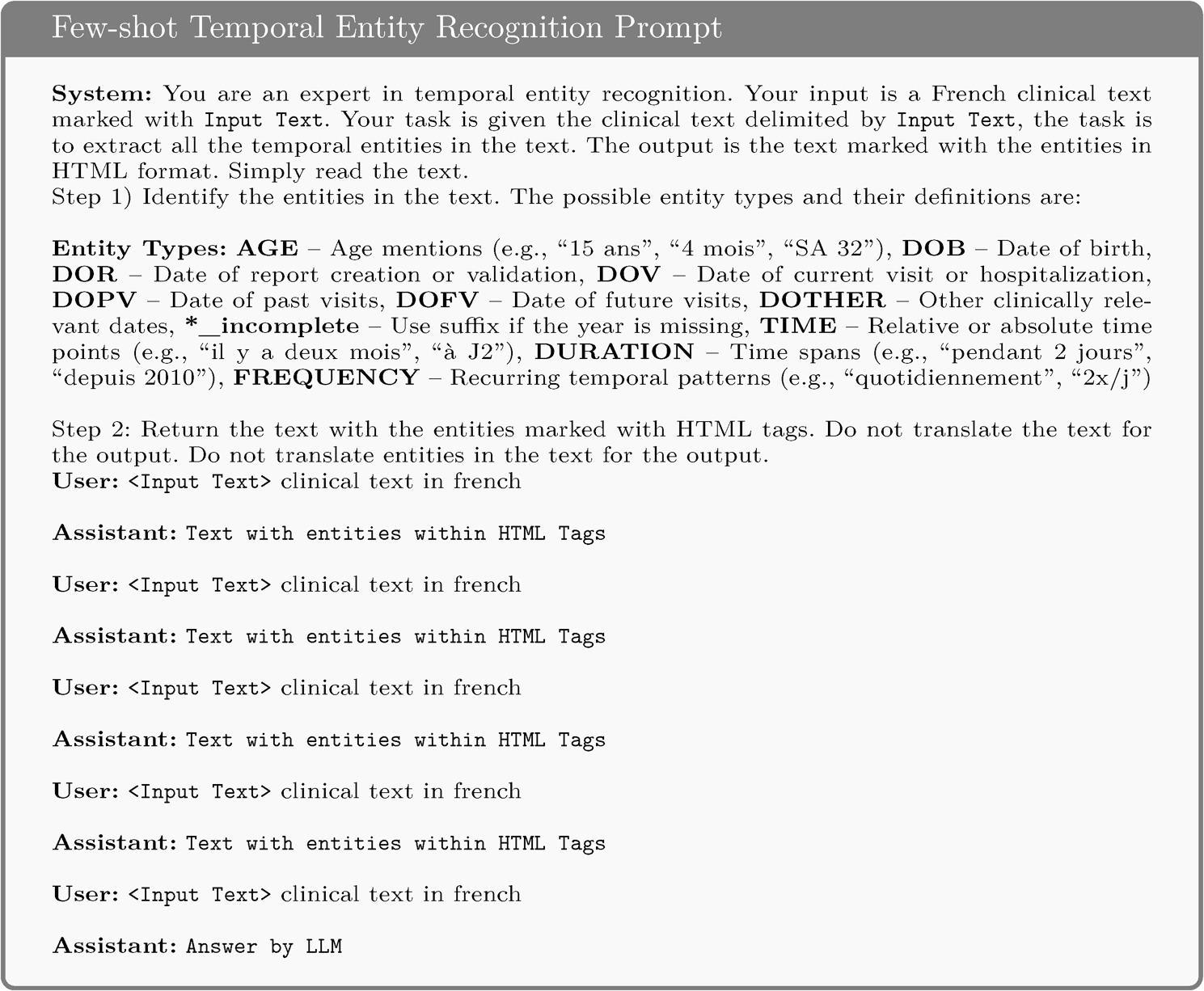


### Temporal relation

To be able to evaluate automatic temporal relation extraction methods, we conducted experiments using the OpenNRE toolkit, Large Language Models, and Parameter Efficient Fine-Tuning with CamemBERT-Bio-Base. For our experiments, we have considered only Time-Phenotypes relations and Phenotype-Time relations as these are primary relations that allow us to construct the timeline of a patient which is our primary objective.

The two-step pipeline is evaluated separately. Table 6 reports Step 1 results (binary classification: does a relation exist between this entity pair?), evaluated on all possible entity pairs in the test set (21,883 ABSENT pairs and 2,408 PRESENT pairs). Table 7 reports Step 2 results (multi-class relation type classification), evaluated only on the gold-standard PRESENT pairs, i.e., pairs confirmed to have a relation. Note that in deployment, Step 2 would receive the output of Step 1—introducing cascading errors.

**Table 6:** Step 1 — Binary relation detection results (does a relation exist between this entity pair?)

	OpenNRE			LLaMA 0-shot			Mistral 0-shot			LLaMA few-shot			Mistral few-shot			PEFT		
**Class**	P	R	F1	P	R	F1	P	R	F1	P	R	F1	P	R	F1	P	R	F1
ABSENT	0.97	0.74	0.84	0.99	0.85	0.92	0.99	0.89	0.94	0.99	0.90	0.94	0.99	0.93	0.96	1.00	0.96	**0.98**
PRESENT	0.07	0.50	0.13	0.17	0.72	0.27	0.23	0.79	0.35	0.24	0.80	0.37	0.35	0.87	0.50	0.52	0.93	**0.67**

Evaluated on all possible entity pairs in the test set: 21,883 ABSENT and 2,408 PRESENT. Best F1 per row in **bold**. Note: PEFT achieves the strongest binary classification performance (PRESENT F1=0.67), substantially outperforming OpenNRE (PRESENT F1=0.13) and both zero-shot and few-shot LLMs (PRESENT F1=0.27–0.50)

#### OpenNRE

Following the default preprocessing strategy adopted in OpenNRE, we selected a fixed window of 100 tokens around the source and target entities. This ensured that the input preserved sufficient contextual cues for temporal relation extraction while remaining compatible with the 512-token limit of CamemBERT-base.

We have used the OpenNRE as our baseline. We have used the “almanach/camembert-base” model for our task. The model is fine-tuned using for two steps of relation extraction separately as explained in the beginning of this Experiments section. The following configuration was used to adapt OpenNRE for CamemBERT-base relation extraction: **model**: camembert-base, batch_size: 32, learning_rate: 2e-5, optimizer: adamw, dropout: 0.1, max_epoch: 10.

**Table 7:** Step 2 — Multi-class temporal relation classification results

		OpenNRE			LLaMA 0-shot			Mistral 0-shot			LLaMA few-shot			Mistral few-shot			PEFT		
Relation	n	P	R	F1	P	R	F1	P	R	F1	P	R	F1	P	R	F1	P	R	F1
BEFORE-OVERLAP	1057	0.68	0.52	0.59	0.81	0.64	0.71	0.85	0.69	0.76	0.88	0.73	0.79	0.92	0.79	0.85	0.94	0.82	**0.87**
BEGINS-AT	739	0.54	0.53	0.54	0.68	0.64	0.66	0.73	0.69	0.71	0.76	0.73	0.74	0.82	0.80	0.81	0.86	0.82	**0.84**
OVERLAP	344	0.51	0.52	0.51	0.63	0.63	0.63	0.67	0.69	0.68	0.74	0.72	0.73	0.81	0.80	0.80	0.82	0.81	**0.82**
CONTAINS	158	0.37	0.54	0.44	0.44	0.62	0.52	0.46	0.68	0.54	0.56	0.70	0.62	0.60	0.78	0.68	0.70	0.80	**0.75**
BEFORE	84	0.30	0.50	0.38	0.35	0.63	0.45	0.46	0.69	0.55	0.45	0.74	0.56	0.54	0.77	**0.64**	0.56	0.80	**0.66**
SIMULTANEOUS	78	0.31	0.54	0.39	0.36	0.63	0.46	0.42	0.71	0.52	0.43	0.72	0.54	0.51	0.78	0.62	0.50	0.79	**0.61**
ENDS-AT	32	0.17	0.53	0.26	0.20	0.62	0.30	0.24	0.66	0.35	0.21	0.96	0.32	0.28	0.76	0.41	0.27	0.78	**0.40**

The evaluation is done on gold-standard PRESENT pairs only. The final column (PEFT end-to- end) evaluates PEFT Step 2 on pairs predicted PRESENT by PEFT Step 1, reflecting realistic deployment performance. Support (n) counts gold-standard instances in the test set. Best gold-evaluation F1 per row in bold. Note: PEFT leads on all seven rela- tion types. The three most frequent relations (BEFORE-OVERLAP, BEGINS-AT, OVERLAP), accounting for over 82% of annotated pairs, all exceed F1=0.82 under PEFT. Performance degrades progressively for lower-frequency relations; ENDS-AT (*n *= 32) remains challenging across all methods, with no approach exceeding F1=0.41. The end-to-end PEFT column reflects realistic deployment performance, where Step 2 operates on Step-1-predicted PRESENT pairs rather than gold-standard pairs, intro- ducing cascading errors that reduce F1 by approximately 0.05–0.10 across relation types

#### Large Language Models (LLM)

To evaluate the performance of LLM in the Temporal Relation Extraction, we used the same two models: LLaMA 8B and Mistral-small 22B, that were used for the Temporal Entity Recognition.

*Zero-shot Prompting:* To assess the models’ ability to infer temporal relations without prior fine-tuning, we employed a structured zero-shot prompting approach. Each prompt included:

- A short excerpt from the clinical narrative (in French)
- a phenotype and an temporal entity which are sure to have a relationship
- A predefined list of possible temporal relation types (see Temporal relationship framework section)
- A direct instruction asking the model to identify the most appropriate relation type between the two entities.

For the binary class classification, the prompt format was designed to simulate a classification task, encouraging the model to select from the two relation types PRESENT and ABSENT.

For the multiclass classification, the prompt format was designed to simulate a classification task, encouraging the model to select from the following relation types: BEFORE, BEFORE_OVERLAP, OVERLAP, SIMULTANEOUS, BEGINS-AT, CONTAINS and ENDS-AT. 
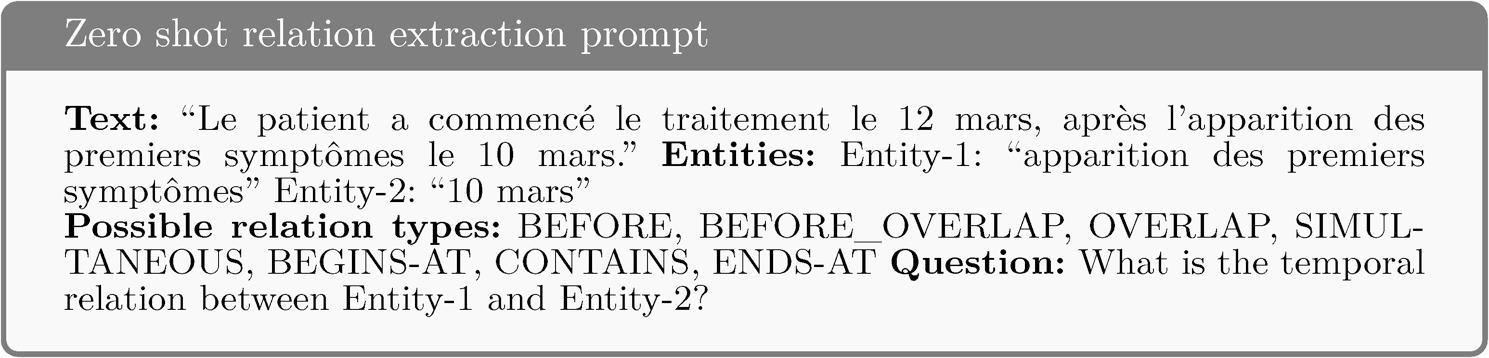


This format was applied uniformly across all test instances, allowing for direct comparison of model predictions against gold-standard annotations.

**Fig. 3:**
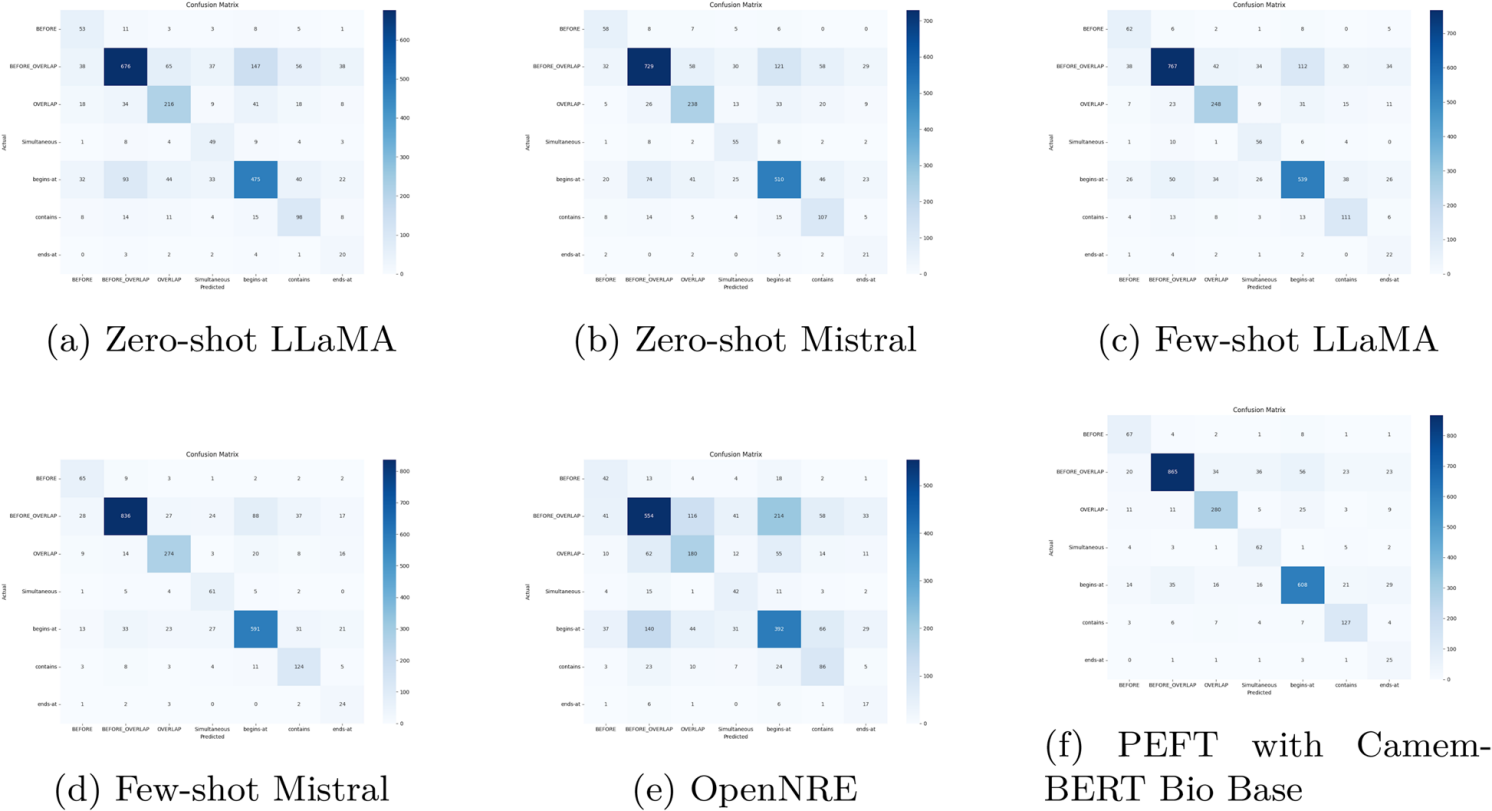
Confusion matrices for temporal relation extraction models — Step 2 multi-class classification on gold-standard PRESENT pairs. Each matrix shows predicted (columns) vs true (rows) relation labels. The diagonal represents correct classifications. OpenNRE (**e**) shows strong bias toward BEFORE-OVERLAP, producing near-zero recall for minority classes. Zero-shot LLMs (**a**, **b**) show more distributed but still imprecise boundaries. Few-shot prompting (**c**, **d**) substantially sharpens diagonal concentration. PEFT (**f**) achieves the clearest diagonal dominance, particularly for the three high-frequency relations. Systematic off-diagonal confusion clusters between semantically adjacent pairs: BEFORE-OVERLAP/BEGINS-AT (both involve temporal precedence) and OVERLAP/SIMULTANEOUS (subtle co-occurrence distinctions). Confusion matrices for named entity recognition are not reported, as token-level NER evaluation is fully summarized by the per-entity precision, recall, and F1 scores in Table 5; confusion matrices would not add interpretive value beyond what is already captured by the support-stratified metrics

**Fig. 4:**
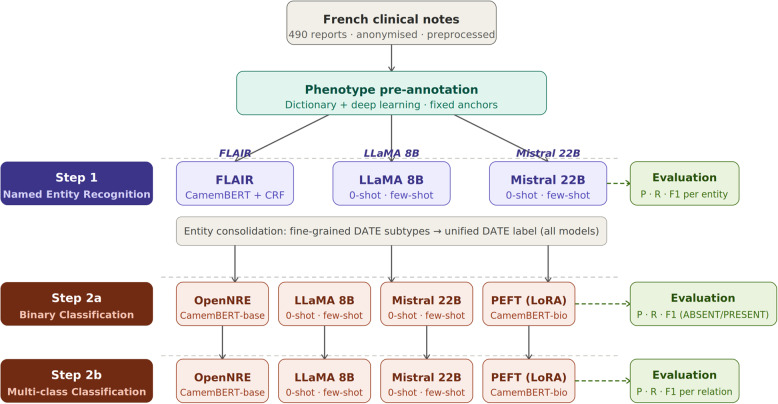
Overview of the experimental pipeline. Clinical notes are first automatically pre-annotated with phenotype spans (Phenotypes pre-annotation section). Step 1 (NER) compares three approaches in parallel—FLAIR with CamemBERT-base, LLaMA 8B, and Mistral 22B—each evaluated under both fine-grained and consolidated DATE entity schemas. Steps 2a–2b (relation extraction) evaluate four approaches on the same three models plus PEFT with LoRA on CamemBERT-bio-base: Step 2a detects the presence or absence of a relation between each entity pair (binary classification); Step 2b classifies the relation type among seven categories (BEFORE-OVERLAP, BEGINS-AT, OVERLAP, CONTAINS, BEFORE, SIMULTANEOUS, ENDS-AT) for pairs identified as related in Step 2a. Each step is evaluated using precision, recall, and F1 individually

*Few shot prompting:* The few-shot prompting strategy was implemented using a structured conversational format, simulating interaction between a system and a language model. Below is an example of the prompt structure used during inference: 
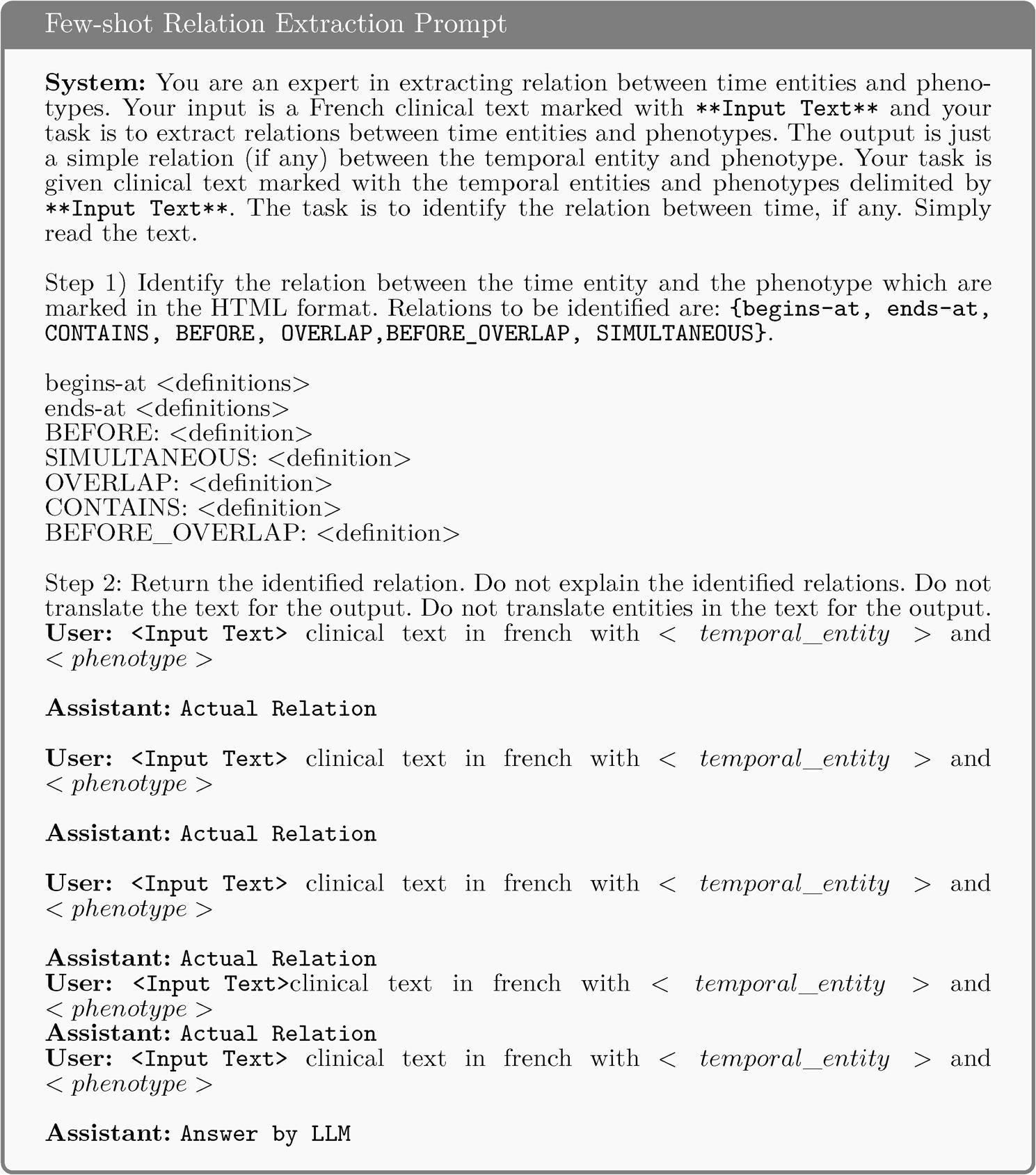


The results of the few-shot approach are summarized in Table 7 and Fig. 3.

#### Fine Tune with PEFT

To be able to use PEFT with CamemBERT-bio-base, we selected a fixed window of 100 tokens around the source and target entities, ensuring sufficient contextual coverage for temporal relation cues while remaining within the 512-token input limit of CamemBERT-bio-base.

Recent advances in Parameter-Efficient Fine-Tuning (PEFT) have demonstrated significant promise for adapting transformer based models, Small and Large language models to specialized tasks such as relation extraction, particularly in low-resource or domain-specific settings like clinical NLP. We applied Parameter-Efficient Fine-Tuning (PEFT), using the HuggingFace’s transformers library, using the LoRA (Low-Rank Adaptation) method on the camembert-bio-base model. The configuration is as follows: base_model: camembert-bio-base, peft_method: LoRA, task_type: SEQ_CLS, lora_alpha: 16, lora_dropout: 0.1, target_modules: [“query”, “value”], training_batch_size: 32, learning_rate: 2e-5, max_epochs: 10.

Table 7 reports multi-class relation classification performance on gold-standard PRESENT pairs; the best F1 per row is shown in bold. PEFT leads on all seven relation types. The three highest-frequency relations (BEFORE-OVERLAP, BEGINS-AT, OVERLAP), which together account for over 82% of annotated pairs, all exceed F1=0.82 under PEFT, while the low-frequency ENDS-AT relation (n=32) remains challenging across all approaches. The confusion matrices for the multi-class classification in Fig. 3.

The prompts presented in this section represent the final versions used for evaluation. Prompt design was guided by informal pilot testing on a small held-out development set; systematic prompt optimization was not conducted, and this constitutes a limitation of the LLM evaluation. Future work should explore prompt sensitivity analysis to quantify the impact of prompt formulation on performance.

## Discussion

### Temporal entity recognition

Our experiments reveal distinct performance profiles across recognition approaches. FLAIR with CamemBERT-base achieved strong performance on well-represented entity types (DOB: F1=0.98, DOR: F1=0.95, Frequency: F1=0.83), demonstrating the effectiveness of supervised sequence labeling for French clinical text. However, FLAIR exhibited severe degradation on incomplete date entities (DOR_incomplete and DOTHER_incomplete: F1=0.00), suggesting that supervised approaches struggle with sparse annotation categories.

Entity consolidation substantially improved performance across all methods. The unified DATE entity achieved F1=0.96 with FLAIR and F1=0.91 with few-shot Mistral, compared to varied performance (F1=0.00–0.95) across fine-grained DATE subtypes. This wide range is driven entirely by class imbalance within fine-grained DATE subtypes. Well-represented subtypes—DOB, DOR, and DOV achieve F1$\ge$ 0.95 with FLAIR because the model encounters sufficient training examples to learn reliable surface patterns. At the opposite extreme, DOR_incomplete and DOTHER_incomplete yield F1=0.00 with near-zero test support, a single missed prediction produces zero recall and a formally undefined or zero F1. This is not a model failure per se, but an artefact of annotation rarity—these subtypes simply do not appear frequently enough in the corpus to support reliable evaluation. The entity consolidation result (unified DATE: F1=0.96) therefore reflects not only a simpler classification task but also the elimination of these unevaluable low-frequency classes. Future work should either collect additional examples of rare subtypes or treat them as a single INCOMPLETE DATE class.This finding suggests that coarser-grained taxonomies may be more suitable for computational approaches while maintaining clinical utility for timeline generation.

Large language models demonstrated modest zero-shot performance but achieved notable improvements with few-shot guidance. Mistral-small 22B consistently outperformed LLaMA 8B, with few-shot Mistral approaching FLAIR’s performance on the consolidated DATE entity. The substantial gap between zero-shot (DATE: F1=0.69–0.80) and few-shot (DATE: F1=0.84–0.91) performance underscores the value of in-context learning for adapting general-purpose models to specialized clinical annotation tasks.

### Temporal relation extraction

Temporal relation classification results demonstrate a clear performance hierarchy: PEFT with CamemBERT-bio-base achieved the strongest overall performance (BEFORE_OVERLAP: F1=0.87, begins-at: F1=0.84, OVERLAP: F1=0.82), followed by few-shot LLMs (Mistral F1=0.80–0.85 for major relations), with OpenNRE substantially underperforming across both classification steps.

#### Binary relation detection

In Step 1 (binary classification), OpenNRE achieved F1=0.84 for ABSENT relations but only F1=0.13 for PRESENT relations, indicating severe class imbalance handling issues. In contrast, zero-shot LLMs performed better (LLaMA: F1=0.27, Mistral: F1=0.35 for PRESENT), with few-shot prompting yielding substantial improvements (LLaMA: F1=0.37, Mistral: F1=0.50). PEFT achieved the strongest binary classification (ABSENT: F1=0.98, PRESENT: F1=0.67), demonstrating robust detection of relation presence.

#### Multi-class relation classification

For Step 2 (multi-class classification), OpenNRE showed limited performance with BEFORE_OVERLAP achieving the highest F1=0.59, followed by begins-at (F1=0.54) and OVERLAP (F1=0.51). The model performed poorly on minority classes: BEFORE (F1=0.38), Simultaneous (F1=0.39), contains (F1=0.44), and ends-at (F1=0.26). This performance gap highlights the limitations of traditional fine-tuning approaches for French clinical temporal relation extraction.

Zero-shot LLMs demonstrated improved performance over OpenNRE, with Mistral achieving F1=0.76 for BEFORE_OVERLAP, F1=0.71 for begins-at, and F1=0.68 for OVERLAP. Few-shot prompting further enhanced performance, with Mistral reaching F1=0.85 for BEFORE_OVERLAP, F1=0.81 for begins-at, and F1=0.80 for OVERLAP—approaching PEFT-level performance on major relation types.

PEFT with CamemBERT-bio-base achieved the strongest results across all relation types, with F1 scores of 0.87 (BEFORE_OVERLAP), 0.84 (BEGINS-AT), 0.82 (OVERLAP), and 0.75 (contains). These three most frequent relations constitute over 82% of annotated relationships, demonstrating PEFT’s effectiveness for the core temporal reasoning required in timeline generation.

#### Confusion matrix analysis

The confusion matrices (Fig. 3) reveal systematic patterns across all models. In binary classification, OpenNRE exhibits the highest false negative rate, frequently misclassifying true relations as absent—explaining its low recall (0.50) despite high precision (0.97) for the ABSENT class. Zero-shot LLMs show improved detection with moderate false positive rates, while few-shot guidance substantially reduces false positives. PEFT demonstrates near-optimal binary classification with minimal errors.

For multi-class classification, the most frequent misclassifications occur between semantically adjacent relations: BEFORE_OVERLAP and BEGINS-AT (both involving temporal precedence with potential continuation), and OVERLAP and Simultaneous (reflecting subtle distinctions in temporal co-occurrence). OpenNRE shows diffuse prediction patterns with strong bias toward BEFORE_OVERLAP, explaining its complete failure on several relation types. The progression from OpenNRE to few-shot LLMs to PEFT demonstrates increasing precision in learned decision boundaries, with PEFT achieving the sharpest diagonal dominance. The ends-at relation remained challenging across all approaches (OpenNRE: F1=0.26, zero-shot LLMs: F1=0.30–0.35, few-shot LLMs: F1=0.32–0.41, PEFT: F1=0.40). Confusion matrix analysis reveals scattered predictions with weak diagonal concentration even for PEFT, directly correlating with the relation’s low frequency (216 instances, 1.73% of corpus). This pattern confirms that severe class imbalance fundamentally limits model performance regardless of architectural sophistication.

### Methodological implications

Our comparative evaluation yields several practical insights for French clinical NLP. First, PEFT strategies offer the optimal balance between performance and computational efficiency, achieving state-of-the-art results (F1=0.82–0.87 for major relations) while requiring substantially fewer trainable parameters than full fine-tuning. The performance gap between PEFT and OpenNRE (both using fine-tuning but with different base models) underscores the critical importance of domain-specific pre-training—CamemBERT-bio’s biomedical specialization provides substantial advantages over general-purpose CamemBERT-base.

Second, few-shot prompting of large language models provides a viable alternative when annotated training data is limited, with Mistral-small 22B approaching PEFT performance on major relation types (F1=0.80–0.85 vs. 0.82–0.87). This finding is particularly relevant for rapid prototyping or deployment scenarios where full model fine-tuning is impractical. However, LLMs struggle with severe class imbalance, as evidenced by their poor performance on ends-at relations.

Third, entity consolidation significantly improves recognition performance without compromising timeline generation capabilities. The unified DATE schema achieves near-identical performance to fine-grained schemas for well-represented categories while dramatically improving performance on rare variants, suggesting that annotation simplification should be prioritized in future guideline development.

### Clinical impact

The current work empirically demonstrates temporal relation extraction achieving F1=0.82–0.87 for core relation types. This constitutes one necessary component of an automated patient timeline system; the full pipeline—including temporal entity normalization, timeline graph construction, conflict resolution, and a user-facing visualization layer—remains future work and has not been evaluated here. We therefore describe the clinical impact of the complete pipeline as a hypothesis, contingent on future development and validation.

If a complete, validated pipeline were deployed, the automated temporal extraction capability demonstrated here could address three clinical challenges: reducing clinician cognitive load in synthesizing fragmented temporal records, enabling faster identification of diagnostic temporal patterns, and supporting retrospective cohort studies by structuring longitudinal data currently locked in narrative text. For rare genetic diseases specifically, where diagnostic delays average 5–7 years, automated temporal pattern recognition has been proposed as a mechanism for earlier diagnosis [3]; however, the quantified impact estimates cited in the literature (e.g., 30–50% reduction in diagnostic delay) reflect projections from AI-assisted rare disease programs broadly, not from temporal NLP systems specifically, and have not been validated for the current framework.

The parameter-efficient nature of PEFT approaches enables deployment on standard institutional computing infrastructure without extensive computational resources. The strong performance with consolidated entity schemas (DATE: F1=0.96) demonstrates that simpler annotation frameworks maintain clinical utility while improving scalability for multi-institutional adoption. For French-speaking healthcare systems with limited NLP resources, this work provides immediately applicable tools to enhance care coordination and enable retrospective cohort studies by structuring temporal information currently locked in narrative text.

### Limitations and future directions

Our study has several limitations. The severe class imbalance for rare relations (particularly ends-at: 1.73% of corpus) limits model performance across all approaches, with even PEFT achieving only F1=0.40. Targeted data augmentation strategies, such as synthetic example generation or active learning focused on minority classes, may help address this limitation. The annotation guidelines, while comprehensive, produced fine-grained DATE distinctions that proved difficult for all models to learn reliably — suggesting that guideline simplification could improve both annotation consistency and model performance. Additionally, evaluation was conducted on a single institution’s data from Necker Hospital, potentially limiting generalizability across different clinical documentation practices, medical specialties, and regional variations in French medical language. Cross-institutional validation would strengthen confidence in the framework’s broader applicability.

Generalization: The annotation guidelines can be adopted without modification by other French clinical institutions, with minimal vocabulary adaptation for institution-specific abbreviations and transfer learning from our trained models. For other languages, the guideline structure and relation taxonomy serve as a structured template, but substantial adaptation effort should be expected: clinical language and jargon differ markedly across languages, and the same caveat applies to existing English frameworks — the THYME corpus guidelines [32] and the I2B2 2012 temporal challenge [33] were not simply translated into French for this work precisely because French clinical temporal expressions, abbreviation systems, and documentation conventions require purpose-built guidelines. Language- and institution-specific validation is necessary before deployment.

Clinical implementation pathway: Integration into existing EHR systems would require secure GDPR-compliant infrastructure, HL7 FHIR interfaces, and prospective validation studies evaluating whether automated timelines reduce synthesis time and improve identification of diagnostically relevant patterns. All outputs would require clinician verification before influencing clinical decisions. The parameter-efficient nature of PEFT enables deployment on standard hospital servers. The temporal extraction framework developed here also enables future investigation of temporal signatures — characteristic patterns of symptom onset, progression, and intervention timing — that may serve as digital biomarkers for rare genetic diseases, potentially supporting differential diagnosis and clinical trial recruitment.

## Conclusion

This study presents a comprehensive framework for temporal relation extraction in French clinical narratives, addressing critical gaps in non-English clinical NLP resources and establishing foundations for digital medicine applications. We developed specialized annotation guidelines tailored to French medical language, producing a high-quality annotated corpus of 490 clinical reports with 12,464 entity-relation pairs and strong inter-annotator agreement (F1 $\ge$ 0.94 for core temporal entities).

Our comparative evaluation demonstrates that parameter-efficient fine-tuning (PEFT) with CamemBERT-bio-base achieves comparable results while maintaining computational efficiency suitable for clinical deployment. Entity consolidation significantly improved recognition across all methods, suggesting that simpler taxonomies enhance both computational performance and annotation consistency. This work advances clinical temporal NLP through three innovations. Methodologically, we demonstrate that PEFT achieves superior performance (F1=0.87 vs. 0.59 for OpenNRE) with the computational efficiency required for clinical deployment. In terms of French clinical annotation, our framework addresses temporal expression patterns specific to French pediatric clinical documentation—including gestational age expressions, relative day-counting conventions such as “J2”, and incomplete date handling—that are not covered by existing French clinical NLP resources such as MERLOT [28] or the oncology-focused annotation presented at [13]. The entity consolidation finding (unified DATE: F1=0.96) provides actionable guidance for future annotation schema design in this domain. Architecturally, our results show that domain-specific pre-training (CamemBERT-bio) outweighs model scale, a finding with direct implications for resource allocation in clinical NLP.

## Data Availability

Due to the highly specific nature of the text data used in this study, and the practical impossibility of guaranteeing complete anonymization at the individual level, French regulations do not allow us to publicly share these data outside the framework of the research agreement, the designated research partners, and the scope of patient consent. Aggregate data, however, are available upon request to the authors. Prior to sharing, a data use agreement must be negotiated between the two parties in order to comply with applicable guidelines and regulations. In accordance with these regulations, the dataset will be archived and stored in a secure enclave. Outside the scope of the current research, access to patient data may be granted only upon submission of a research project to a duly authorized scientific and ethics committee. The process can be initiated by contacting dpd@institutimagine.org.
